# Interface Microstructure and Properties of Vacuum-Hot-Rolled 55#/316L Clad Rebars

**DOI:** 10.3390/ma16020571

**Published:** 2023-01-06

**Authors:** Zhen Li, Zecheng Zhuang, Xuehai Qian, Yong Xiang, Lei Zeng, Jianping Tan

**Affiliations:** 1 School of Mechanical and Electrical Engineering, Central South University, Changsha 410083, China; 2 Hunan Provincial Engineering Research Centre for Laminated Metal Composites, Changsha 410083, China; 3 Technology Centre, Guangxi Liuzhou Iron and Steel Group Ltd., Liuzhou 545002, China; 4 Hunan Santai New Materials Ltd., Loudi 417000, China

**Keywords:** clean-interface assembly, second-phase particles, metallurgical bonding

## Abstract

The existing process for the preparation of cladded rebars is too complicated for large-scale industrial production. Therefore, this paper proposes a 55#/316L rebar preparation method based on vacuum hot rolling. The microstructure and mechanical properties of the composite interface of the rebar, along with the connecting technique, were studied using transmission electron microscopy, X-ray diffraction, and Vickers hardness testing. The obtained results showed that the minimum thickness of the 55#/316L rebar cladding was 0.25 mm, which was twice that of the M 329M/M 329-11 design standard used in the United States of America. Due to the diffusion of carbon, large numbers of second-phase particles were precipitated on the stainless-steel side, which resulted in intergranular chromium depletion. After multi-pass hot rolling, the minimum bonding strength of the composite interface reached 316.58 MPa, which was considerably higher than the specified value of 210 MPa. In addition, we designed three different types of rebar connection joints: sleeve, groove-welded, and bar-welded. According to the tensile test, the bar-welded joint had higher yield strength (385 MPa) and tensile strength (665 MPa) than the base rebar (376.6 MPa and 655 MPa), as well as a very high corrosion resistance.

## 1. Introduction

Rebar is widely used in building materials, and it is in significant demand [1,2,3]. In 2021, the world’s total steel output reached 1.951 billion tons, of which rebar accounted for approximately 18% [4]. Ordinary rebars are prone to corrosion and expansion. Therefore, concrete structures can be damaged, and building life is severely reduced when the volume increases beyond the elastic limit of concrete [5,6,7]. In addition, roads must be maintained, which has high costs. For example, according to the Federal Highway Administration, road maintenance costs more than USD 20 billion per year in the United States of America, and the annual maintenance cost for bridges in Germany is in the range of EUR 6–30 billion [8]. In order to effectively improve the safety performance and service life of roads, bridges, and important buildings, two methods have been used that prevent and delay the corrosion of reinforcing bars in such structures. The protective ability of building structures can first be improved by increasing the thickness of the concrete. However, this method significantly increases the cost of the concrete structures [9]. Protective layers have also been added to the surface of rebar by spraying epoxy resin powder or zinc plating, for example. However, the coatings are prone to damage and corrosion during transportation and construction [10,11,12,13]. Clad rebars use two different metal-rolled composite compositions. The rebar outer layer is composed of stainless steel, which has very high corrosion resistance, while the base metal is carbon steel, which meets the relevant mechanical property requirements. In addition, clad rebar has a lower price than stainless rebar. Several studies have been conducted on the optimization of the rolling process and the preparation technology for clad rebars. For instance, Szota et al. [14] established the constitutive equation for the inner and outer layers of C45/306L rebar based on the Tresca yield criterion, and a numerical simulation of the finished hole and non-finished hole was conducted using Forge2005 software (Material Forming Research Center, Paris, France). The results showed that the thickness distribution of the steel cladding rolled with the flat elliptical pass was more uniform than that of the single-radius and multi-radius ellipses. Sawicki et al. [15,16] placed stainless steel rods below the non-melting electrode and used tungsten inert gas-shielded welding to generate the high temperature needed to melt the stainless steel rods and spray onto the carbon steel surface. However, this process was limited by certain issues, such as the uneven distribution of the stainless steel cladding and the difficulty of engaging the rolling mill. Feng et al. [17] used a thermal simulator to prepare 20MnSi/306L rebars with different compression temperatures (950 °C and 1050 °C) and compression rates (50% and 70%). When the compression rate increased from 50% to 70%, the number of oxides on the composite interface significantly decreased, the grains became continuously thinner, and the tensile strength of the rebar increased from 589 MPa to 690 MPa. When the compression temperature increased, significant amounts of C elements crossed the composite interface to reach the stainless-steel side and reacted with Cr to produce carbon–chromium compounds, which resulted in an increase in the hardness value of the stainless steel near the composite interface. All these findings were based on thermal simulators, and whether they can guide industrial production should be verified. The American company STELAX recycled wasted iron scraps and fused them into rod shapes. They were then placed into a stainless steel pipe, which was subsequently sealed at the end face and then rolled. The South Dakota Department of Transport deduced from the performance test of its product that there were large holes between the clad and the base, which indicated that the iron scraps and stainless steel pipe could not undergo effective bonding during the hot rolling [18]. Pak et al. [19] used electroslag welding technology to prepare a composite billet of rebar. Stainless steel powder was first placed on the surface of the carbon steel. Resistance heat generated by the slag was then used to melt the stainless steel powder to form a preliminary bond between the stainless steel and carbon steel. However, the production efficiency of the assembly process was low, and significant amounts of impurities were present in the composite interface of the blank. Liu et al. [20] used a flux deposition process to prepare a 20MnSiV/Cr13 composite billet. The 20MnSiV surface was first polished. The Cr13 stainless steel was then heated until liquefaction. Afterwards, the Cr13 was sprayed onto the carbon steel surface using a special nozzle. Although this process effectively solved the interface oxidation problem affecting the billet during the heating and rolling, the operation is more complicated, and it is still in the laboratory stage. Xie et al. [21] first pulled a carbon steel rod and stainless steel pipe so that the inner and outer layers of the metal produced a certain pretension force, and the billet was then sealed and rolled with six passes. The experiment results showed that the clad of the cladded rebar was evenly distributed, with all the mechanical properties meeting the relevant requirements. However, this process requires a pulling process and supporting facilities to be added in the existing rebar production line, which reduces the production efficiency. Our team successfully rolled out 20MnSiV/316L rebar in Liuzhou Iron and Steel Plant with vacuum-hot-rolling technology in 2021. Through testing, it was found that the tensile properties, bonding properties, and bending properties of the 20MnSiV/316L rebar met the CNS GB/T 36707-2018 standard, and it was close to 316L stainless steel in terms of its corrosion-resistance properties, which is ten times as high as carbon rebar [22].

This paper presents a preparation method for 55#/316L rebar that can be used to simplify the preparation process while guaranteeing the properties of the clad rebar, as well as providing experience and a reference to help scholars select materials for the inner and outer layers of rebar. This process involves inserting the carbon steel after surface treatment into a stainless steel pipe. Welding and sealing are then performed and, finally, hot rolling is conducted for shaping. In 2021, the as-produced clad rebars were successfully applied in the construction of the Quanhe Bridge in Linquan, which was the first application of its kind in China.

## 2. Materials and Methods

### 2.1. Rebar Materials

In this study, 55# steel, which has high strength and wear resistance, was used. It is a type of medium carbon steel, and it was selected as the base metal for steel reinforcement. In addition, 316L stainless steel was selected as the clad material for the rebar, since its corrosion resistance is close to that of 2205 stainless steel, while its cost is lower. The chemical compositions of the base and cladding materials are presented in Table 1.

### 2.2. Assembly Process for the 55#/316L Rebar

**Figure 1:**
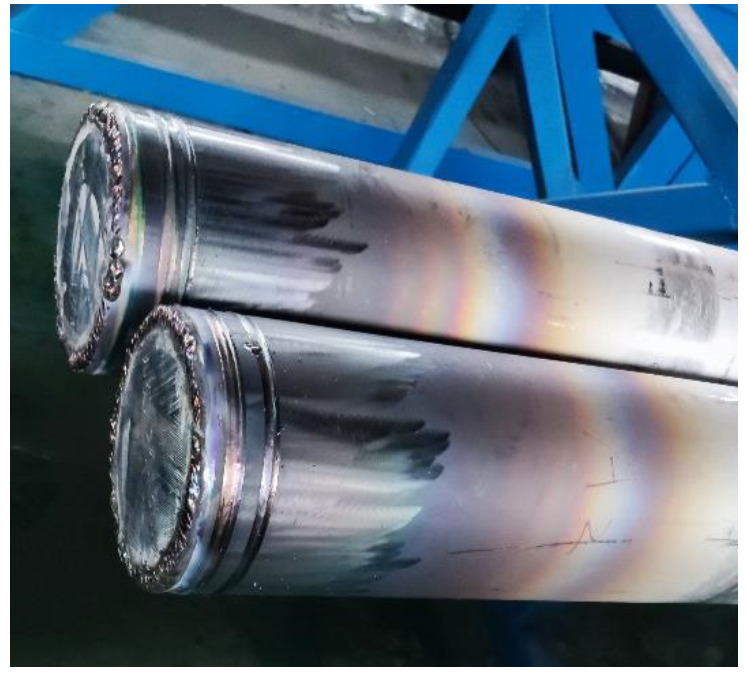
Appearance and morphology of the composite billet.

To reduce the formation of inclusions on the composite interface and achieve effective metallurgical bonding of the clad rebars, a 55#/316 rebar formation process based on the clean-interface assembly was used. The 55# steel rod was first machined to the specified size of Φ147.2 mm × 9000 mm. The burrs were then removed from the surface of the carbon steel rod using a hand-held grinder. Afterwards, a hydraulic machine was used to push the carbon steel rod into the stainless-steel pipe. Finally, the billet was vacuumed and end-welded [22]. A schematic of the composite billet end-seal is shown in Figure 1. The specifications and size of the composite billet are presented in Table 2.

### 2.3. Rolling Process for 55#/316L Rebar

The rolling was performed on the no. 6 bar production line of Liuzhou Iron and Steel Plant. The production line consists of 18 mills, among which the 9th, 10th, 15th, and 16th mills were not functional. The steps of the 55#/316L rebar rolling process were as follows. The composite billet was first heated in a three-staged heating furnace, and the billet was then rolled using a two-roller mill. The rolling speed of the K1 mill was 48 m/s.

**Figure 2:**
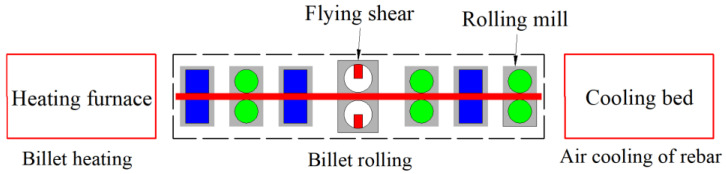
Rolling process for the 55#/316L rebar.

In order to prevent the temperature of the head and tail of the billet from being very low and thus difficult to engage in the next mill, flying shears were used to cut off the head and tail of the billet after rough rolling and medium rolling, respectively. The rolling process for the 55#/316L rebar is shown in Figure 2, and the rolling parameters are presented in Table 3.

### 2.4. Experimental Method

A rebar sample with a length of 10 mm was produced by cutting with a wire-cutting machine, dividing the rebar into four parts along the end surface, and then grinding and polishing a quarter round surface with 240#, 400#, 600#, 1000#, 1200#, and 1500# sandpapers and linting in succession. The rebar base was then etched and cladded with the mixed solution with volume ratios of HNO_3_:C_2_H_6_O = 3:97 and HCl:C_6_H_3_N_3_O_7_:C_2_H_6_O = 5:1:100, respectively. After etching, the rebar was placed under a BH200M metallographic microscope (Olympus Corporation, Tokyo, Japan) for observation.

In this study, 180#, 400#, 800#, and 1200# sandpapers were used to polish the end face and axial surface of the rebar. After grinding, a GD2500M-H industrial camera (Doshen Technology Co., Ltd., Shenzhen, China) was used to capture the images of the end face and axial surface of the rebar in order to observe whether the distribution of cladding was uniform.

An EPMA-8050G electron probe (Shimadzu, Tokyo, Japan) was used for the line and surface scanning of the composite interface of the rebar at an accelerating voltage of 15 kV. A HVT-10Z Vickers hardness tester (Jinan Huayin Test Instrument Co., Jinan, Shandong, China) was used to test the hardness of the composite interface with a test load of 500 g.

The mechanical properties of the rebar were tested using a WW-1000-G electro-hydraulic servo universal testing machine (Sinheng Technology Co., Ltd., Shenzhen, China). The tensile length of the rebar was 500 mm, and no treatment was performed on it before the test. The fracture morphology of the rebar was studied using a TESCAN VEGA3 scanning electron microscope (Tescan, Brno, South Moravia, Czech Republic).

A DX-2700BH diffractometer (Dandong Haoyuan Instrument Co., Dandong, Jilin, China) was used to detect the types of second-phase particles on the composite interface with a test range of 20–90° and a test speed of 3°/min.

The binding strength of the rebar was tested based on the method presented in [22]. The shear sample was first placed in a shear fixture, and the cladding was then punched and sheared from the carbon steel using the indenter of a universal test machine. Finally, the bonding strength of the rebar was calculated based on the punching and shearing force and the area of the composite interface.

Based on the procedure specified by the GB/T10125-2012 Chinese standard, a corrosion test was conducted on the rebar joints, and rust removal was undertaken after the corrosion test. The corrosion test parameters are presented in Table 4.

## 3. Results and Discussions

### 3.1. Surface Contour Analysis

**Figure 3:**
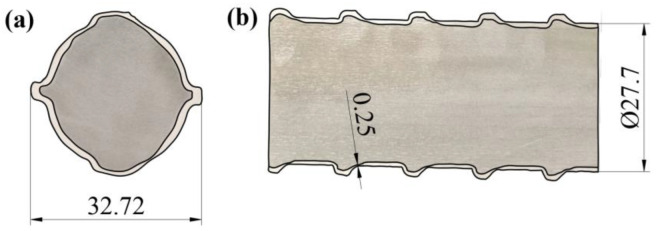
Cross-section images of the 55#/316L rebar (wt. mm): (**a**) end surface and (**b**) axial surface.

Cross-section images of the 55#/316L rebar are shown in Figure 3. No defects, such as leakage points, were observed on the end face or axial surface of the rebar. After measurements, it was deduced that the inner diameter of the rebar was 27.7 mm, and the longitudinal rib height was 2.51 mm. Note that these values met the M 329M/M 329-11 American design standard. In addition, the minimum thickness of the cladding of the 55#/316L rebar was 0.25 mm, which was approximately twice the designed value.

### 3.2. Metallographic Structure Analysis

**Figure 4:**
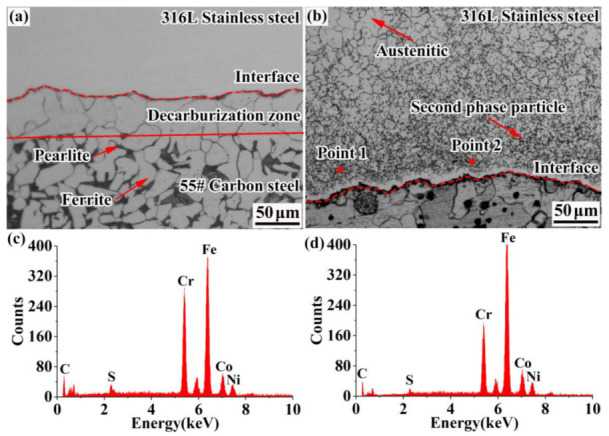
Metallographic images of the rebar composite interface: (**a**) carbon-steel side, (**b**) stainless-steel side, (**c**) element distribution at point one, and (**d**) element distribution at point two.

Metallographic images of the composite interface of the 55#/316L rebar are shown in Figure 4. It can be seen that the composite interface was not straight, which was due to the fact that the yield strength of the 55# steel was almost 110 MPa under a rolling temperature of 1100 °C, much lower than that of the 316L stainless steel (200 MPa). Therefore, under the same load, the base material would undergo excessive deformation, resulting in asynchronous deformation of the metals on the two sides of the composite interface and further leading to plastic instability [23,24].

The black material on the carbon-steel side was pearlite, and the gray material was ferrite. A lower amount of pearlite was present at regions closer to the composite interface. This was mainly because the carbon content of the 55# steel (0.53%) was significantly higher than that of the 316L stainless steel (0.021%), and the large concentration difference provided a sufficient driving force for the diffusion of carbon. Therefore, carbon would spread to the cladding layer during the rolling process. Thus, the carbon content of the base material and the formation of cementite and pearlite were reduced.

**Figure 5:**
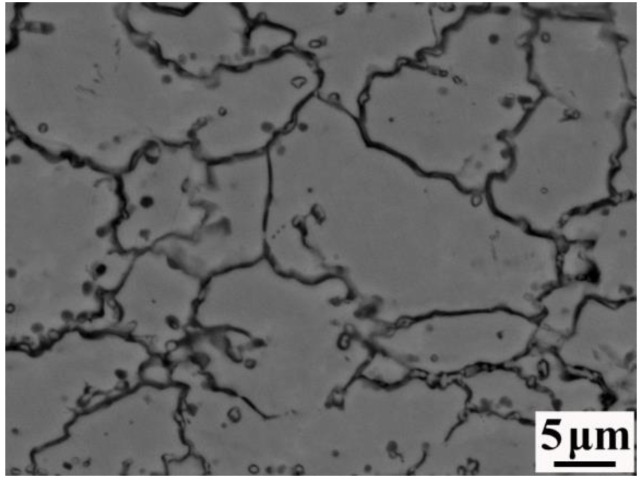
SEM image of the 55#/316L reinforcement stainless-steel side.

A large number of small and dense second-phase particles were continuously distributed on the stainless-steel side. It can be seen from Figure 5 that the second-phase particles were mainly distributed along the grain boundary. This was because carbon has a lower weight, smaller volume, and faster diffusion rate than Mn, Si, and other elements. Therefore, carbon more rapidly crossed the composite interface to the stainless-steel side and reacted with Cr, Si, Mo, and other elements to generate second-phase particles during the rolling process. In order to determine the element composition of the second-phase particles, an electron probe was used to point-sweep them. The results of the point scanning are presented in Table 5. The second-phase particles were mainly composed of C, S, Cr, Fe, Co, and Ni, while the contents of S, Co, and Ni were very low, accounting for 1.9%, 1.7%, and 2.2%, respectively. Therefore, it was deduced that the second-phase particles were carbides. Mas et al. [25] studied dissimilar 18MND5/309L joints. They deduced that a large number of chromium-rich carbides appeared in the stainless steel grains, which is consistent with the findings of this study.

### 3.3. Phase Analysis of the Carbide Material Using X-ray Diffraction (XRD)

**Figure 6:**
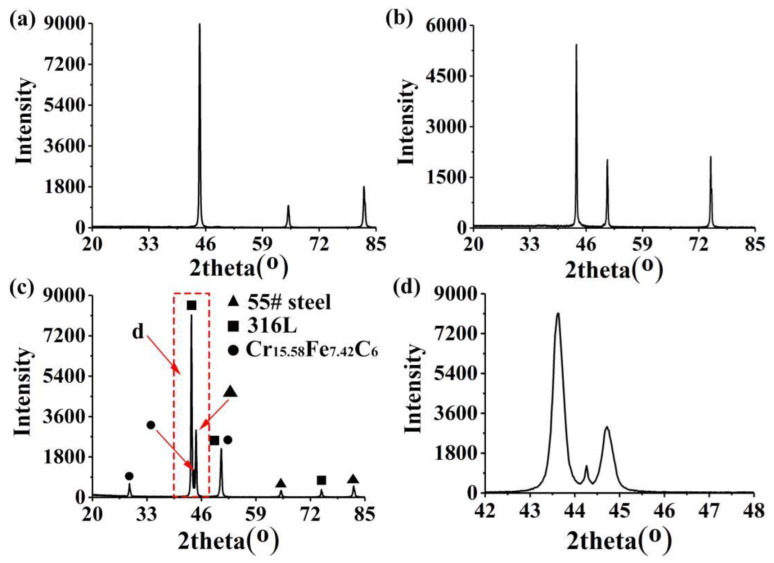
XRD results: (**a**) 55# steel, (**b**) 316L stainless steel, (**c**) 55#/316L rebars, and (**d**) partial enlarged view of XRD results.

To determine the specific chemical formula for the second-phase particles, XRD analysis was performed on the 55# steel, 316L stainless-steel, and 55#/316L rebars. The diffraction results are shown in Figure 6. For the 55#/316L rebar, eight peaks were observed. The results were consistent with the XRD database. The diffraction results for the 55# steel and 316L stainless steel indicated that the fourth, sixth, and eight (from left to right) peaks were due to the 55# steel, and the second, fifth, and seventh peaks belonged to the 316L stainless steel. In addition, the first, third, and fifth peaks were due to Cr_15.58_Fe_7.42_C_6_. Therefore, the chemical formula for the second-phase particles could be determined as Cr_15.58_Fe_7.42_C_6_. Jiang et al. [26] detected large amounts of carbides in the carburized layer of a stainless-steel composite plate using transmission electron microscopy and determined the type of the carbides to be M_23_C_6_ through the calibration of the diffraction signals of the carbides.

### 3.4. Electron Probe Analysis of the Composite Interface

**Figure 7:**
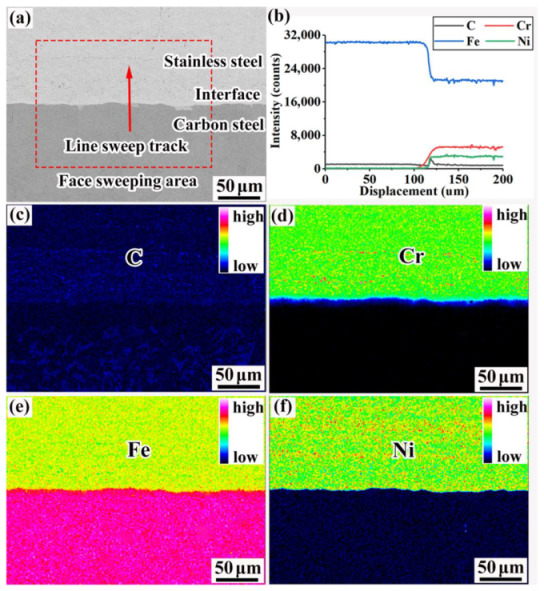
Line and surface scanning results for each element of the composite interface: (**a**) line-sweep track and surface-sweep area, (**b**) line scanning results for each element [30], (**c**) surface scanning results for C, (**d**) surface scanning results for Cr, (**e**) surface scanning results for Fe, and (**f**) surface scanning results for Ni.

The surface scanning and line scanning results for the composite interface are shown in Figure 7. The composite interface of the 55#/316L rebar was very clean, without any inclusions. This was due to the fact that the composite interface was vacuumed during the assembly of the billets, which reduced the amount of residual oxygen. The reduction rate for the 55#/316L rebar was as high as 83%. During large deformations, the oxide on composite interfaces is continuously crushed and fully dissolved into the base and coating [27].

Due to the different contents of each element in the base metal and cladding, C, Fe, Cr, and Ni diffusion occurred during the rolling process. The order of elements, sorted based on the diffusion distance, was as follows: C (~80 µm) > Cr (~45 µm) > Fe (~35 µm) > Ni (~15 µm). The diameter of carbon was only 154 pm, which was significantly smaller than that of Fe, Cr, and Ni. Therefore, gap diffusion of carbon occurred in the base and cladding material [28]. In the interstitial solid solution, the energy required by the diffusion of solute atoms was smaller than that in the substitutional solid solution. Therefore, the diffusion speed was higher. Liu et al. [29] deduced differences in the concentration gradient and diffusion coefficient of C, Cr, and Ni in a Q235/304 composite plate, and the diffusion distances were, respectively, 50–60, 10–12, and 5–6 µm, as estimated using surface and line scanning [29].

### 3.5. Hardness Analysis of the Composite Interface

**Figure 8:**
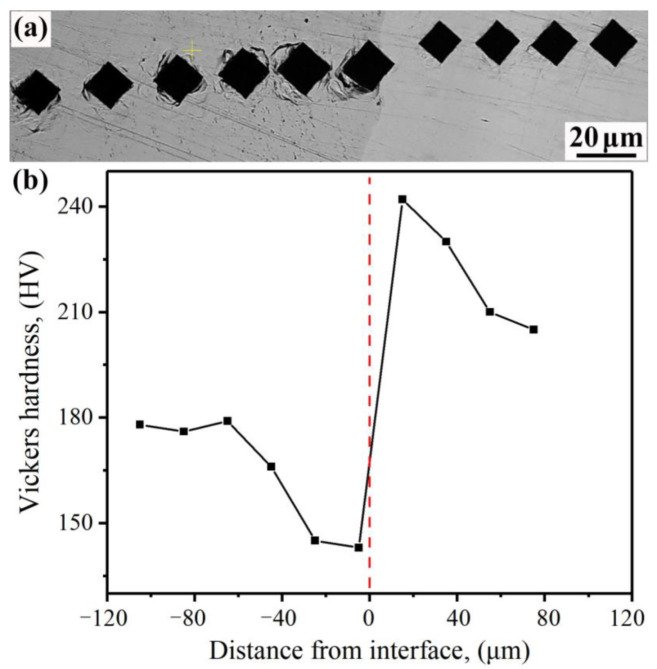
Hardness distribution of the rebar composite interface: (**a**) locations of test points and (**b**) microhardness.

The hardness distribution of the composite interface of the 55#/316L rebar is shown in Figure 8. It can be seen that, from the carbon-steel side to the stainless-steel side, the hardness decreased at the beginning and then increased. The minimum value of approximately 143 HV was adjacent to the composite interface of the carbon-steel side, and the maximum value of almost 242 HV appeared near the composite interface of the stainless-steel side. The change in hardness was closely related to the properties of the base and cladding materials. As the pearlite content near the composite interface of the carbon steel decreased, the microhardness value also decreased. On the stainless-steel side, carbon reacted with chromium to form a large number of brittle second-phase particles, and the hardness value increased. In addition, at greater distances from the composite interface, fewer second-phase particles were present. Therefore, the hardness values eventually tended to stabilize. It is important to mention that the hardness value of the 55# steel matrix (~176 HV) was lower than that of the 316L stainless steel (~205 HV). This was due to the fact that, compared to the 55# steel, the deformation of the cladding stainless steel was greater and the degree of work hardening was higher.

### 3.6. Analysis of the Stretching Performance

The tensile properties of the 55#/316L rebar were tested three times. The rebar tensile test results are presented in Table 6. The average values for the yield strength, tensile strength, and ductility were 378.3 MPa, 651.6 MPa, and 14.2%, respectively.

**Figure 9:**
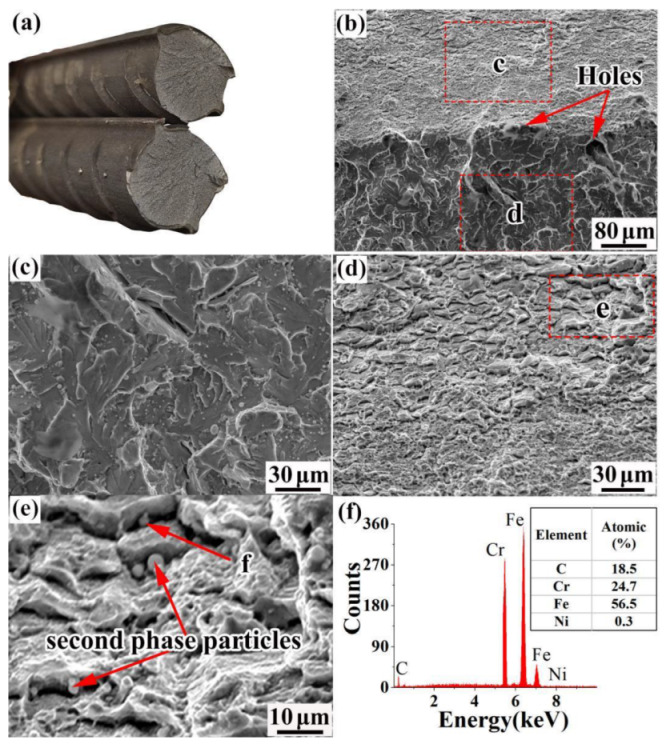
Tensile fracture morphology of the 55#/316L rebar: (**a**) macroscopic morphology, (**b**) microstructure of the composite interface, (**c**) tensile morphology of the stainless-steel sides, (**d**) tensile morphology of the carbon-steel sides, (**e**) particle distribution of the second-phase particles, and (**f**) chemical composition of the second-phase particles.

The tensile fracture morphology of the 55#/316L rebar is shown in Figure 9. It can be seen from Figure 9a that the section color of the 55#/316L rebar was bright, the fracture was perpendicular to the stress direction, and no shear lips could be observed. Figure 9b shows the morphology of the composite interface of the fracture. Several holes could be observed in the composite interface with diameters in the range of 5–10 µm. Certain differences in attributes existed between the 55# steel and 316L stainless steel. During stretching, deformation occurred inconsistently, leading to an axial tearing force in the composite interface. When the bonding force of the composite interface was weaker than the axial tearing force, holes were formed. Figure 9c shows the tensile morphology of the carbon-steel side. A certain number of cleavage steps and river-like patterns appeared on the carbon-steel side. Figure 9d shows the tensile morphology of the stainless-steel side, which presented a typical intergranular fracture. It can be seen from Figure 9e,f that the main reason for this phenomenon was that a large number of second-phase particles precipitated at the grain boundary of the stainless steel. The second-phase particles were carbides, which reduced the intergranular bonding force of the stainless steel. Under the action of tension, cracks were formed in the carbide as a priority and propagated along the grain boundary. Hong et al. [31] studied the delayed fracture behavior of three kinds of twin-induced plastic (TWIP) steels. They deduced that when the intergranular carbide content increased, the fracture resistance of the specimens decreased.

### 3.7. Analysis of the Bonding Strength of the Composite Interface

The results of the bonding strength test are presented in Table 7. It can be seen that the minimum bonding strength of the composite interface was 316.58 MPa, which was higher than the specified value of 210 MPa. This indicated that an effective metallurgical bonding of the 55#/316L rebar was achieved after multi-pass hot rolling.

**Figure 10:**
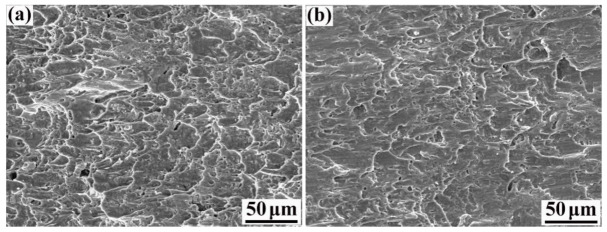
Shear fracture morphology of rebar: (**a**) carbon-steel side and (**b**) stainless-steel side.

The shear fracture morphology of the 55#/316L rebar is shown in Figure 10. In contrast to the tensile fracture, the shear fracture was mainly caused by shear stress. Therefore, the fracture dimples were parabolic.

More dimples were present on the carbon-steel side than the stainless-steel side. These observations can be explained by two main factors: (1) the organization of carbon steel near the composite interface was mainly ferritic, and ferrite has a higher toughness than austenite; (2) due to the diffusion of carbon, a large number of second-phase particles precipitated on the stainless-steel side. The hardness and brittleness of the second-phase particles were significantly higher than those of the stainless-steel matrix, which resulted in a higher stress concentration under the action of shear stress and further caused the stainless-steel side to undergo brittle fracturing.

### 3.8. Bending Performance Analysis

**Figure 11:**
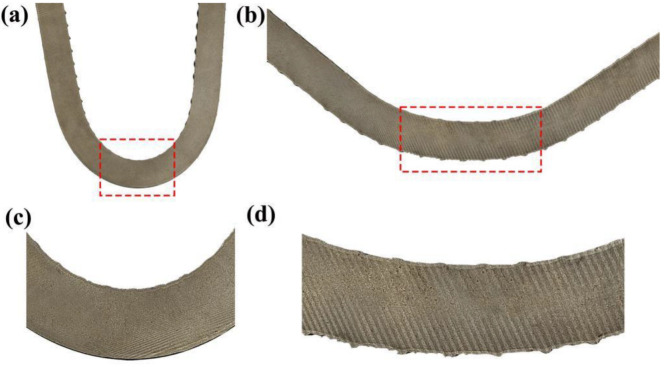
Schematics of the rebar bending profile: (**a**) positive bending, (**b**) reverse bending, (**c**) partial enlarged view of positive bending, and (**d**) partial enlarged view of reverse bending.

To accurately observe the combination of composite interfaces after the positive and reverse bending of the rebar, it was cut along the axis. Profile diagrams of the positive and reverse bending of the 55#/316L rebar are shown in Figure 11. It can be seen that the composite interface did not separate after the positive and reverse bending of the rebar.

### 3.9. Studies of the 55#/316L Rebar Connection Technology

The mechanical properties and corrosion resistance of joints directly affect the safety of concrete structures. Three kinds of joints were fabricated in this study: sleeve, groove-welded, and bar-welded.

**Figure 12:**
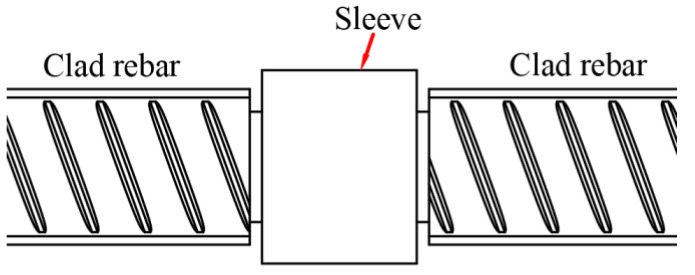
Diagram of the rebar sleeve connection.

The connection diagram of the rebar sleeve is shown in Figure 12. A lathe was used to cut threads (M22 × 2.5) on the end face of the rebar, and the latter was screwed into the sleeve.

**Figure 13:**
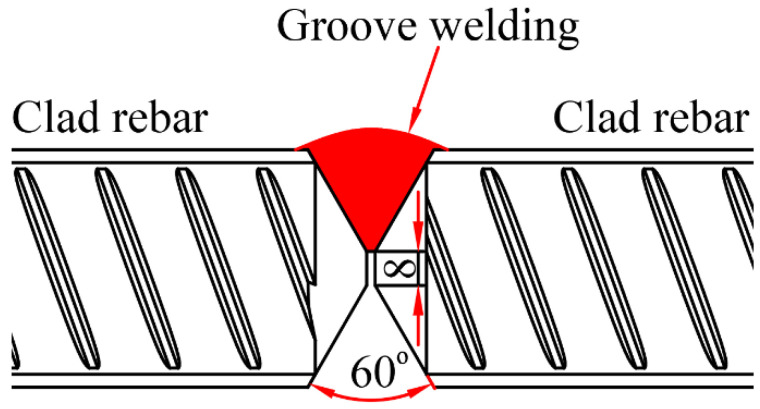
Schematic of the rebar groove welding.

A schematic of the rebar groove welding is shown in Figure 13. The groove was first machined on the rebar end. The groove was oriented 60° from the horizontal direction, and the rebar end was welded using a torch.

**Figure 14:**
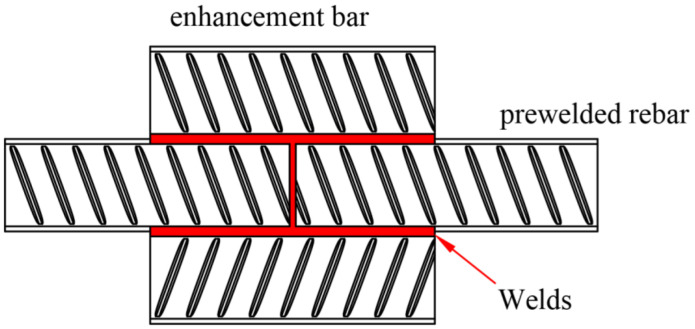
Schematic of the rebar bar welding.

A schematic of the bar welding is shown in Figure 14. The rebars to be welded were initially placed head to head, and gaps between the two rebar end faces were almost 2 mm. An enhancement bar was then placed on the two sides of the prewelded rebar. The length of the bar was approximately 140 mm. In addition, to improve the mechanical properties of the welded joint, double-sided welding of the bar was conducted. The welding process parameters for rebar are presented in Table 8.

**Figure 15:**
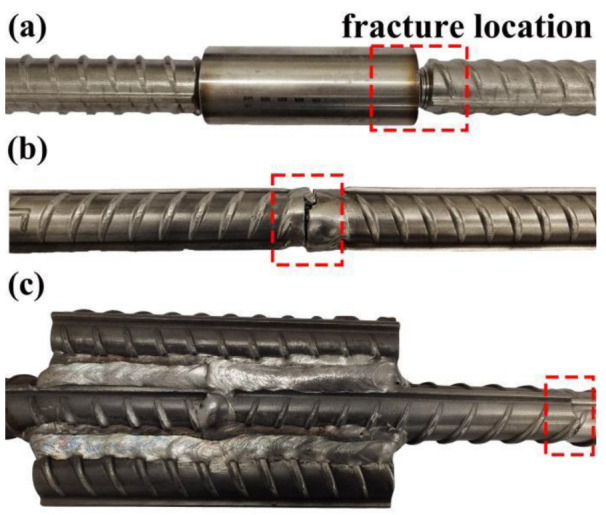
Fracture position for each rebar: (**a**) sleeve connection, (**b**) groove-welding connection, and (**c**) enhancement bar-welding connection.

Afterwards, the tensile properties of the as-fabricated three joints were tested. Note that each joint was tested three times. The fracture positions of the rebars are shown in Figure 15. For the rebars connected via sleeve and groove welding, the fracture positions occurred on the joint, while for the rebars connected via bar welding, the fracture position was located in the base metal. Table 9 shows the tensile test results for the three types of joints. It can be seen that the tensile properties of the three types of joints were stable. The yield strength and tensile strength of the bar-welding joint were, respectively, 385 MPa and 660 MPa, which were higher than those of the base metal (378.3 MPa and 651.6 MPa, respectively). However, the tensile strengths of the sleeve and groove-welded joints were lower than that of the base metal.

**Figure 16:**
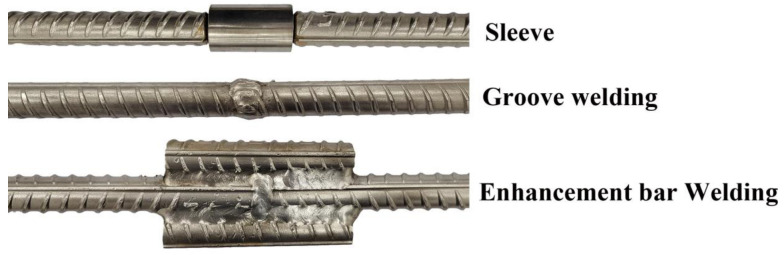
Surface morphology of each joint after rust removal.

**Figure 17:**
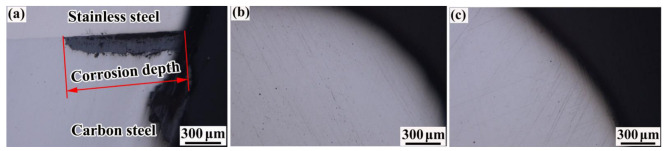
Corrosion depth of each joint after rust removal: (**a**) corrosion depth of sleeve, (**b**) corrosion depth of the bar-welded joint, and (**c**) corrosion depth of the groove-welded joint.

A neutral salt spray corrosion test was performed with an exposure time of 30 d. Figure 16 and Figure 17 show the surface morphology and corrosion depth of each joint after rust removal, respectively. Significant corrosion was observed at the connection between the sleeve and the rebar. The corrosion depth was almost 1.04 mm, which was caused by the presence of receding grooves on the surface of the rebar. This prevented effective sealing between the sleeve and the rebar. In addition, the corrosion depth of the composite interface was significantly deeper than that of the carbon steel matrix. The reason for this phenomenon was that a large amount of chromium-rich carbides precipitated from the composite interface of the rebar, which resulted in a chromium-poor composite interface and reduced the corrosion resistance. Therefore, the corrosion cracks preferentially spread along the composite interface. No small cracks were observed in the groove-welded joint or the bar-welded joint after 30 d of exposure, which indicated that the groove-welded joint and the bar-welded joint had very high corrosion resistance.

## 4. Conclusions

1. The 55#/316L clad rebar was prepared using a clean-interface assembly and vacuum-hot-rolling processes. The dimensions of the rebar were consistent with the M 329M/M 329 design standard of the United States of America, and the minimum thickness of the rebar cladding was 0.25 mm, which was twice the design value;
2. As a result of the diffusion of carbon, a large number of second-phase particles precipitated on the stainless-steel side. The chemical formula for the second-phase particles, determined by XRD, was Cr_15.58_Fe_7.42_C_6_;
3. The composite interface of the 55#/316L rebar was very clean and without any inclusions, which was mainly because of the vacuum treatment of the billet and the high rolling reduction. The order of elements, sorted based on the diffusion distance, was: C (~80 µm) > Cr (~45 µm) > Fe (~35 µm) > Ni (~15 µm);
4. The minimum bonding strength of the composite interface was 316.58 MPa, which was higher than the specified value of 210 MPa. This indicated that the 55#/316L rebar achieved effective metallurgical bonding after multiple passes of hot rolling;
5. Three different rebar joints were designed. Compared with the sleeve and groove-welded joints, the bar-welded joints had better mechanical properties and higher corrosion resistance;
6. Compared with the 20MnSiV/316L rebar, the carbide on the stainless-steel side of the 55#/316L reinforcement obviously increased, the bonding property of the composite interface decreased, and the corrosion-resistance property was reduced.

## Figures and Tables

**Table 1:** Chemical composition of the 55# steel and 316L stainless steel (wt.%).

Material	C	Si	Mn	P	S	Cr	Ni	Mo	Fe
55# steel	0.53	0.28	0.55	0.011	0.006	0.19	0.04	/	Bal.
316L	0.021	0.65	1.81	0.018	0.008	17.9	13.5	2.1	Bal.

**Table 2:** Specifications and size of the billet.

	316L Stainless Steel Tube	55# Steel Rod
Size (mm)	Φ159 × 6 × 9000	Φ147.2 × 9000

**Table 3:** Rolling parameters for the 55#/316L rebar.

Heating Temperature (°C)	Heating Time (h)	Rolling-Mill Type	Rolling Speed (m/s)	Cooling Method
1250–1280	3	Two-roller	48	Air cooling

**Table 4:** Test parameters for salt spray corrosion.

Test Type	Corrosion Time (d)	Corrosion Box Temperature (°C)	Rust Removal Temperature (°C)	Rust Removal Solution
Neutral salt spray corrosion test	30	35	60	4 L pure nitric acid + 20 L distilled water

**Table 5:** Point-sweep results for second-phase particles.

	C	S	Cr	Fe	Co	Ni
Spot 1	18.5	1.9	30	45.7	1.7	2.2
Spot 2	16.2	0.7	26.5	52.2	1.9	2.5

**Table 6:** Results of the 55#/316L rebar tensile experiment.

Serial Number of Rebar	Yield Strength (MPa)	Tensile Strength (MPa)	Ductility (%)	Average Values (MPa; MPa; %)
1	375	650	14.3	378.3; 651.6; 14.2
2	380	650	14.3	
3	380	655	14.0	

**Table 7:** Test results for the bonding strength (wt. MPa).

Type	Maximum Value	Minimum Value	Average Value	Standard Deviation
55#/316L rebar	334.96	316.58	326.1	6.27

**Table 8:** Welding process parameters for rebar.

Welding Type	Welding Material	Welding Speed (mm/s)	Welding Height (mm)
Groove welding	316L	3–5	5–8
Enhancement bar welding	316L	3–5	5–8

**Table 9:** Results of the tensile test for each joint (wt. MPa).

Joint Type	Yield Strength	Tensile Strength	Standard Deviation of Tensile Strength
Sleeve	/	432	1.52
Groove-welded	/	585	2.89
Enhancement bar-welded	385	660	2.89
